# Demographic, Clinical, and Mycologic Differences Between Invasive Fungal Sinusitis and Pneumonia in Hematologic Malignancy

**DOI:** 10.1093/ofid/ofag613

**Published:** 2026-09-26

**Authors:** Simran Gupta, Tejas S Athni, Esther Arbona-Haddad, Inia Perez Villa, Alexis Liakos, Alexandra Tong, Maria Shehata, Carolyn Strauch, Muneerah M Aleissa, Alice Z Maxfield, Regan W Bergmark, Victor Kovac, Amy C Sherman

**Affiliations:** Division of Infectious Diseases, Brigham and Women's Hospital, Boston, Massachusetts, USA; Dana-Farber Cancer Institute, Boston, Massachusetts, USA; Harvard Medical School, Boston, Massachusetts, USA; Harvard Medical School, Boston, Massachusetts, USA; Division of Infectious Diseases, Brigham and Women's Hospital, Boston, Massachusetts, USA; Division of Infectious Diseases, Brigham and Women's Hospital, Boston, Massachusetts, USA; Division of Infectious Diseases, Brigham and Women's Hospital, Boston, Massachusetts, USA; Division of Infectious Diseases, Brigham and Women's Hospital, Boston, Massachusetts, USA; Division of Infectious Diseases, Brigham and Women's Hospital, Boston, Massachusetts, USA; Harvard Medical School, Boston, Massachusetts, USA; Department of Medicine, Beth Israel Deaconess Medical Center, Boston, Massachusetts, USA; Division of Infectious Diseases, Brigham and Women's Hospital, Boston, Massachusetts, USA; College of Pharmacy, Department of Pharmacy Practice, Princess Nourah Bint Abdulrahman University, Riyadh, Saudi Arabia; Harvard Medical School, Boston, Massachusetts, USA; Department of Otolaryngology, Brigham and Women's Hospital, Boston, Massachusetts, USA; Harvard Medical School, Boston, Massachusetts, USA; Department of Otolaryngology, Brigham and Women's Hospital, Boston, Massachusetts, USA; Dana-Farber Cancer Institute, Boston, Massachusetts, USA; Harvard Medical School, Boston, Massachusetts, USA; Division of Infectious Diseases, Massachusetts General Hospital, Boston, MA, USA; Division of Infectious Diseases, Brigham and Women's Hospital, Boston, Massachusetts, USA; Dana-Farber Cancer Institute, Boston, Massachusetts, USA; Harvard Medical School, Boston, Massachusetts, USA

**Keywords:** hematologic malignancy, invasive fungal disease, invasive fungal pneumonia, invasive fungal sinusitis, neutropenia

## Abstract

Among 132 people with hematologic malignancies and invasive fungal disease, invasive fungal sinusitis was associated with greater dissemination, lower biomarker sensitivity, more diverse fungal pathogens, and higher rates of chronic rhinosinusitis and sinonasal defects than invasive fungal pneumonia. These findings suggest distinct clinical syndromes with different risk factors and diagnostic considerations.

Invasive fungal disease (IFD) is a major cause of morbidity and mortality in patients with hematologic malignancies (HMs) [1]. Established risk factors for IFD include prolonged neutropenia, corticosteroid use, graft-versus-host disease (GVHD), and relapsed or refractory malignancy [2, 3]. Following inhalation of fungal conidia, clinical disease manifests most commonly as either invasive fungal pneumonia (IFP) or invasive fungal sinusitis (IFS) [1, 4]. However, while these 2 presentations share a common pathogenic origin, they differ in their clinical presentation, microbiology, management, and outcomes, and factors influencing site of disease remain poorly understood.

Prior studies suggest that sinus involvement is significantly more frequent in mucormycosis than in invasive aspergillosis, while pulmonary disease predominates in aspergillosis [1, 5, 6]. Despite these observations, most epidemiologic studies have examined IFD as a composite endpoint or focused on a single anatomic site, and direct comparative analyses of host-level risk factors between IFS and IFP are lacking [3, 6]. We therefore evaluated demographic, clinical, and mycologic differences between people with IFS and IFP to better characterize factors associated with site of presentation.

## METHODS

We conducted a retrospective, case–case analysis of adults with HM and IFS or IFP at a tertiary-care academic center [7]. Invasive fungal sinusitis cases met European Organisation for Research and Treatment of Cancer/Mycoses Study Group Education and Research Consortium (EORTC/MSGERC) criteria for proven disease; IFP cases met criteria for proven or probable disease [8]. A positive Mucorales polymerase chain reaction (PCR) assay was considered to meet microbiological criteria. Invasive fungal sinusitis cases diagnosed between 2005 and 2025 were compared with IFP cases diagnosed between 2014 and 2024. Patients were identified from preexisting clinical databases at Brigham and Women's Hospital and Dana-Farber Cancer Institute [7]. Additional variables were collected through detailed electronic medical record (EMR) review. Eligible patients were adults ≥ 18 years of age with HM, IFD, and available EMR data. This study was approved by the Mass General Brigham Institutional Review Board.

Patient demographics, laboratory results at IFD diagnosis, oncologic history, risk factors including recent upper or lower respiratory tract infection within 60 days (diagnosed by respiratory viral swab or clinical syndrome), known sinonasal anatomical defects, preexisting lung disease, prior sinus surgery, chronic rhinosinusitis, nasal steroids or decongestants, inhaled corticosteroids, mycologic characteristics, and mortality outcomes were collected.

Preexisting sinus or pulmonary disease was defined as either (1) documentation of a chronic sinus or lung condition in the EMR or (2) cross-sectional imaging obtained more than 60 days before the diagnosis of IFD demonstrating chronic anatomic changes—specifically nasal polyps, septal deviation, sinonasal cysts, bronchiectasis, emphysema, or interstitial lung disease. These structural findings are categorically distinct from the radiologic features required to satisfy the EORTC/MSGERC clinical criterion for probable disease (well-circumscribed lesions with or without a halo sign, air-crescent sign, cavity, or wedge-shaped/segmental or lobar consolidation in the lung and evidence of invasive sinus infection, such as bony erosion) [9]. Consequently, preexisting sinus or pulmonary disease would not have contributed the imaging findings used to classify a case as probable IFD. Disseminated fungal disease was defined as fungal involvement of 2 or more noncontiguous anatomic sites, as evidenced by radiographic, histopathologic, or microbiologic findings.

Neutropenia was defined as <500/µL and hypogammaglobulinemia as IgG < 300 mg/dL. For the 1,3-beta-D-glucan assay, reference ranges were <60 pg/mL negative; 60–79 pg/mL indeterminate; and ≥80 pg/mL positive. For the galactomannan assay, an index < 0.5 was considered negative, and ≥0.5 positive. Mucorales PCR testing was performed using the ARUP Laboratories assay. Lack of documentation was interpreted as an absence of a finding.

Continuous variables were compared using Mann–Whitney *U* tests and categorical variables using χ^2^ or Fisher's exact tests. Univariable and multivariable analyses identified risk factors associated with an increased risk of IFS compared with IFP. To improve the comparability of the cohorts, a sensitivity analysis restricted to people with proven IFS or IFP between 2014 and 2024 was conducted. Analyses were conducted using R v4.6.0.

## RESULTS

Twenty-eight cases of IFS (all proven) and 104 cases of IFP (22 proven, 82 probable) met inclusion criteria. Age and sex distribution were similar between cohorts (Table 1). Acute myeloid leukemia (AML) was the most common underlying disease and was more common among people with IFP than IFS (88.5% vs 64.3%). Hematopoietic cell transplant (HCT) rates were similar among people with IFP and IFS (53.8% vs 50.0%). The prevalences of diabetes (19.2% vs 21.4%) and hypogammaglobulinemia (32.7% vs 46.4%) were also similar between cohorts. While known parenchymal lung disease (31.7% vs 32.1%) and recent upper or lower respiratory tract infection (21.2% vs 21.4%) were similar among people with IFP and IFS, chronic rhinosinusitis (3.9% vs 17.9%) and preexisting sinonasal anatomical defects (13.5% vs 32.1%) were significantly more prevalent in patients with IFS. There were no differences between cohorts regarding recent oncologic treatments, medications, or GVHD rates.

**Table 1.** Baseline Characteristics

Characteristics^a^	Invasive Fungal Sinusitis (n = 28)	Invasive Fungal Pneumonia (n = 104)	*P* Value^b^
Demographics			
Age at IFD diagnosis (y), median (IQR)^c^	59 (52–66)	62 (52–69)	.51
Time from HM diagnosis to IFD (d), median (IQR)	196 (38.5–1229.8)	334 (102.8–733.8)	.47
Male, n (%)	15 (53.6)	66 (63.5)	.34
Hematologic malignancy, n (%)			
Acute myeloid leukemia	18 (64.3)	92 (88.5)	.**008**
Acute lymphoblastic leukemia	0 (0.0)	2 (1.9)	1.00
Chronic lymphocytic leukemia	0 (0.0)	3 (2.9)	1.00
Multiple myeloma	2 (7.1)	1 (1.0)	.11
Myelodysplastic syndrome	0 (0.0)	1 (1.0)	1.00
Other	4 (14.3)	5 (4.8)	.10
Myelofibrosis	1 (3.6)	0 (0.0)	.21
Non-Hodgkin lymphoma	3 (10.7)	0 (0.0)	.**009**
Hematopoietic cell transplantation, n (%)	14 (50.0)	56 (53.8)	.72
Allogeneic	13 (46.4)	57 (54.8)	.43
Autologous	1 (3.6)	1 (1.0)	.38
Comorbidities, n (%)			
Diabetes mellitus	6 (21.4)	20 (19.2)	.80
Underlying lung disease	9 (32.1)	33 (31.7)	.97
Emphysema	0 (0.0)	7 (6.7)	.34
Asthma	3 (10.7)	6 (5.8)	.40
Bronchiectasis	5 (17.9)	12 (11.5)	.36
Interstitial lung disease	0 (0.0)	3 (2.9)	1.00
COPD	1 (3.6)	5 (4.8)	1.00
Hypogammaglobulinemia	13 (46.4)	34 (32.7)	.18
Viral URI or LRI in preceding 60 d	6 (21.4)	22 (21.2)	.97
Chronic rhinosinusitis	5 (17.9)	4 (3.9)	.**02**
Sinonasal anatomical defect^d^	9 (32.1)	14 (13.5)	.**046**
Prior sinus surgery	3 (10.7)	3 (2.9)	.11
Medications, n (%)			
Intravenous immunoglobulin	2 (7.1)	21 (20.2)	.16
Nasal steroids or decongestants	4 (14.3)	13 (12.5)	.76
Steroid inhaler	5 (17.9)	16 (15.4)	.77
Oncologic treatment, n (%)			
Systemic steroids	8 (28.6)	39 (37.5)	.38
Antimetabolites	24 (85.7)	79 (76.0)	.27
T-cell suppressants	2 (7.1)	3 (2.9)	.29
GVHD in the last 60 d, n (%)	4 (14.3)	34 (32.7)	.06

Table 1 depicts the demographics, comorbidities, and oncologic status of people with invasive fungal pneumonia (IFP) and invasive fungal sinusitis (IFS).

Bold values are *P* values that met level of significance.

Abbreviations: IQR, interquartile range; IFD, invasive fungal disease; HM, hematologic malignancy; COPD, chronic obstructive pulmonary disease; URI, upper respiratory tract infection; LRI, lower respiratory tract infection; GVHD, graft-versus-host disease.

^a^Percents for subcategories are reported as a percentage of the overall total N patients, and not of the total in the subcategory in which they nested.

^b^ *P* values were computed for continuous variables using the Mann–Whitney *U* test and for categorical variables using Fisher's exact test (if at least 1 cell had an expected count of <5) or χ² test.

^c^A rescaled age variable was used in the logistic regression model to compute the odds ratio for a 10-y increase in age.

^d^Sinonasal anatomical abnormalities included septal deviation, nasal polyps, and sinonasal cysts.

Localized disease was more common in IFP (89.4% vs 50.0%), whereas disseminated disease was more common in IFS (10.6% vs 50.0%) (*P* < .0001) (Table 2). Similar proportions of patients with IFP and IFS were neutropenic or hypogammaglobulinemic at the time of fungal diagnosis. Breakthrough fungal infection rates were low and similar (7.7% vs 7.1%, *P* = .92) [10].

**Table 2.** Clinical, Laboratory, and Microbiological Features of Invasive Fungal Sinusitis Versus Invasive Fungal Pneumonia

Characteristics^a^	Invasive Fungal Sinusitis (n = 28)	Invasive Fungal Pneumonia (n = 104)	*P* Value^b^
Spread of infection, n (%)			
Localized	14 (50.0)	93 (89.4)	**<**.**0001**
Disseminated	14 (50.0)	11 (10.6)	**<**.**0001**
Lab values at the time of IFD diagnosis			
Neutropenia, n (%)	22 (78.6)	71 (68.3)	.29
Any absolute neutrophil count < 500 cells/μL in the last 6 m, n (%)	20 (71.4)	86 (82.7)	.18
IgG (mg/dL), median (IQR)	645 (560–853)	646 (390–1020)	.94
Azole prophylaxis at the time of diagnosis, n (%)	2 (7.1)	8 (7.7)	.92
Fluconazole	2	5	
Voriconazole	0	2	
Posaconazole	0	1	
Isavuconazole	0	0	
Serum (1,3)-ß-D-glucan, n (%)			
Positive	6 (21.4)	51 (49.0)	.**01**
Negative	22 (78.6)	53 (51.0)	
Galactomannan index, n (%)			
Positive	2 (7.1)	42 (40.4)	.**001**
Negative	24 (85.7)	62 (59.6)	
Mucorales PCR, n (%)			
Positive	2 (7.1)	9 (8.7)	1.00
Negative	4 (14.3)	16 (15.4)	
Unknown	22 (78.6)	79 (76.0)	
Fungal pathogen, n (%)^c^			
*Aspergillus* species	6 (21.4)	80 (76.9)	**<**.**0001**
*Curvularia* spp.	2 (7.1)	1 (1.0)	.11
Mucorales	6 (21.4)	14 (13.5)	.37
*Fusarium* spp.	5 (17.9)	4 (3.9)	.**02**
*Scedosporium* spp.	1 (3.6)	1 (1.0)	.38
Hyaline mold, species not identified	1 (3.6)	4 (3.8)	1.00
Dimorphic fungi	0 (0.0)	0 (0.0)	1.00
Other	1 (3.6)	1(1.0)	.51
No observable species^d^	6 (21.4)	3 (2.9)	.**003**
Time from IFD diagnosis to last follow-up (d), median (IQR)	272 (26–646)	88 (22–664)	.51
Time from IFD diagnosis to death (d), median (IQR)	76 (20–303)	68 (11–248)	.72
Overall mortality at the end of follow-up, n (%)	20 (71.4)	85 (81.7)	.23

Table 2 depicts the mycological and laboratory characteristics, as well as outcomes of invasive fungal pneumonia (IFP) and invasive fungal sinusitis (IFS) patients.

Bold values are *P* values that met level of significance.

Abbreviations: IQR, interquartile range; PCR, polymerase chain reaction; IFD, invasive fungal disease.

^a^Percents for subcategories are reported as a percentage of the overall total *N* patients, and not of the total in the sub-category in which they nested.

^b^ *P* values were computed for continuous variables using the Mann–Whitney *U* test and for categorical variables using Fisher's exact test (if at least 1 cell had an expected count of <5) or χ² test.

^c^Multiple fungal species were identified for select patients, including *Aspergillus*/Mucorales for n = 2 and *Aspergillus*/*Fusarium* for n = 1; percentage sums thus exceed 100%.

^d^No observable species defined as fungal forms on histopathology but no species able to be identified.

*Aspergillus* spp. was the most common pathogen overall and predominated in IFP cases (76.9% vs 21.4%, *P* < .0001), while *Fusarium* spp. was more common in IFS (3.9% vs 17.9%, *P* = .02) (Table 2). There were also differences in fungal markers between cohorts, with serum 1,3-B-D-glucan (49.0% vs 21.4%, *P* = .01) and galactomannan (40.4% vs 7.1%, *P* .001) more often positive in IFP cases, but no difference in Mucorales PCR positivity (8.7% vs 7.1%, *P* = 1.00). The sensitivity of galactomannan for *Aspergillus* spp. was 41/80 (51.3%) in IFP and 1/6 (16.7%) in IFS, with positive predictive values (PPVs) of 41/42 (97.6%) and 1/2 (50.0%), respectively. The sensitivity of 1,3-B-D-glucan overall for fungal disease was 51/104 (49.0%) in IFP and 6/28 (21.4%) in IFS. The sensitivity of the Mucorales PCR for mucormycosis was 9/13 (69.2%) in IFP and 2/6 (33.3%) in IFS, with a PPV of 100% for both.

One-year all-cause mortality was high in both cohorts: 71/104 (68.3%) in IFP and 16/28 (57.1%) in IFS, with 26/71 (36.6%) deaths in the IFP cohort and 10/16 (62.4%) deaths in the IFS cohort attributed to fungal infection.

On multivariable analysis (Supplementary Table 1), chronic rhinosinusitis and known sinonasal anatomical defects were significant risk factors for the development of IFS over IFP ([OR 5.69, 95% CI 1.23–27.24] and [OR 5.20, 95% CI 1.69–16.43], respectively), with prior sinus surgery trending toward significance (OR 5.44, 95% CI .88–33.92, *P* = .058). Conversely, lung disease and recent respiratory tract infection were not risk factors for IFP versus IFS.

On sensitivity analysis restricted to proven IFP and IFS cases diagnosed between 2014 and 2024 (Supplementary Table 2), sinonasal anatomical defect was again a risk factor for the development of IFS over IFP (OR 14.38, 95% CI 1.34–154.26) (Supplementary Table 3).

## DISCUSSION

In this retrospective analysis of 132 patients with HM and IFD, we identified distinct clinical and mycologic differences between IFS and pneumonia. Chronic rhinosinusitis and sinonasal anatomical defects—and possibly prior sinus surgery—were independently associated with the development of IFS over IFP, suggesting that local anatomic factors may influence the site of invasive mold disease. Traditional systemic risk factors were similarly distributed between cohorts and did not differentiate sinus from pulmonary disease [3]. In contrast, preexisting lung disease and recent respiratory tract infections were not associated with IFP, suggesting that local anatomic predisposition may be particularly important in sinus disease.

The mycologic differences further support that IFS and IFP represent distinct clinical syndromes. *Aspergillus* spp. predominated in IFP, consistent with prior epidemiologic studies [1, 11]. In contrast, IFS demonstrated a more diverse mycologic spectrum, including a significantly greater proportion of *Fusarium* spp. This enrichment of non-*Aspergillus* molds in sinus disease aligns with prior observations that fusariosis and mucormycosis disproportionately involve the sinuses [1, 6]. The higher rate of disseminated disease observed in IFS underscores the severity of sinus presentations and their propensity for local extension [12, 13].

Our findings regarding serum fungal biomarkers have important diagnostic implications. Serum galactomannan sensitivity was markedly lower in IFS than in IFP, consistent with prior studies demonstrating reduced diagnostic accuracy in invasive *Aspergillus* sinusitis [14]. Similarly, 1,3-β-D-glucan positivity was lower in IFS, and the Mucorales PCR—while maintaining 100% PPV in both cohorts—had reduced sensitivity in IFS. These findings suggest that clinicians should not rely on negative serum biomarkers to exclude IFS and reinforce the importance of tissue biopsy for diagnosis [2].

Our study has several limitations. First, the retrospective, single-center design and modest sample size limit generalizability and power for subgroup analyses. Second, IFS cases were limited to proven disease, whereas IFP cases included proven and probable disease. Third, the cohorts spanned different time periods, introducing potential temporal confounding related to evolving antifungal prophylaxis, diagnostic modalities, and treatment paradigms. In particular, the progressive adoption of mold-active azole prophylaxis over these intervals—notably posaconazole (FDA approved in 2006 for prophylaxis of invasive *Aspergillus* and *Candida* infections) and later isavuconazole (approved in 2015 for treatment of invasive aspergillosis and mucormycosis), supplanting earlier fluconazole-and voriconazole-based regimens, has been associated with a shift in the isolated fungal spectrum away from *Aspergillus fumigatus* and *Candida albicans* toward nonfumigatus *Aspergillus* spp., Mucorales, and other rare, frequently azole-resistant molds, which may have differentially affected the pathogen distribution observed between the 2 cohorts [15, 16]. However, our institution does not recommend routine antimold prophylaxis for people with AML or those undergoing HCT; thus, these changes in prophylaxis over time were unlikely to have substantially influenced our findings. Additionally, sensitivity analyses restricted to proven disease during overlapping time periods yielded similar results, suggesting that these differences were unlikely to influence the identified risk factors. Fourth, classifying absent documentation as absence of a clinical finding may have led to underascertainment of some risk factors. Finally, cohort construction may have resulted in incomplete capture of some non-Mucorales, non-*Fusarium* IFP cases outside people with AML, which could have influenced the observed distribution of less common mold species.

In conclusion, this study represents one of the first direct comparative analyses of host-level risk factors between IFS and IFP in people with HMs. The identification of chronic rhinosinusitis and sinonasal anatomical defects as independent predictors of sinus disease may have implications for risk stratification and surveillance, particularly in people with known sinonasal pathology who develop febrile neutropenia. However, specific risk factors for IFP were not identified, highlighting diagnostic challenges and the need for improved risk factor characterization. Future multicenter studies are needed to validate these findings and determine targeted screening approaches in high-risk patients to facilitate earlier diagnosis and improve outcomes.

## Supplementary Material

ofag613_Supplementary_Data

## Supplementary Data

Supplementary materials are available at *Open Forum Infectious Diseases* online. Consisting of data provided by the authors to benefit the reader, the posted materials are not copyedited and are the sole responsibility of the authors, so questions or comments should be addressed to the corresponding author.
