# Supplementary material for: Demographic, Clinical, and Mycologic Differences Between Invasive Fungal Sinusitis and Pneumonia in Hematologic Malignancy
Source: Open Forum Infect Dis. 2026 Sep 26;13(10):ofag613. doi: 10.1093/ofid/ofag613 (PMC13650480; doi:10.1093/ofid/ofag613)
Supplement: Supplementary file 1 [file ofag613_supplementary_data.docx]

**Supplemental Content**

**Demographic, Clinical, and Mycologic Differences Between Invasive Fungal Sinusitis and Pneumonia in Hematologic Malignancy**

Table of Contents

[eTable 1	2](#_Toc231035049)

[eTable 2	3](#_Toc231035050)

[eTable 3	5](#_Toc231035051)

# eTable 1

Age-adjusted, univariate, and multivariable-adjusted logistic regression results for select risk factors differentiating invasive fungal sinusitis from invasive fungal pneumonia.

| Characteristic | Univariable Odds Ratio (95% CI) ^1^ | P-value | Age-Adjusted Odds Ratio (95% CI) ^2^ | P-value | Multivariable-Adjusted Odds Ratio (95% CI) ^3^ | P-value |
| --- | --- | --- | --- | --- | --- | --- |
| Underlying lung disease | 1.01 (0.41 – 2.46) | 0.97 | 1.01 (0.41 – 2.49) | 0.98 | 1.10 (0.40 – 3.00) | 0.86 |
| Viral URI or LRI | 1.02 (0.37 – 2.81) | 0.97 | 1.03 (0.37 – 2.88) | 0.96 | 1.17 (0.38 – 3.54) | 0.79 |
| Chronic rhinosinusitis | **5.43 (1.35 – 21.84)** | **0.02** | **5.43 (1.35 – 21.84)** | **0.02** | **5.69 (1.26 – 25.73)** | **0.02** |
| Sinonasal anatomical defect | **3.01 (1.14 – 7.97)** | **0.046** | **3.13 (1.15 – 8.47)** | **0.03** | **5.20 (1.69 – 16.04)** | **0.004** |
| Prior sinus surgery | 4.04 (0.77 – 21.23) | 0.11 | 4.04 (0.77 – 21.22) | 0.10 | 5.44 (0.94 – 31.46) | 0.06 |

Abbreviations: CI, confidence interval; URI, upper respiratory infection; LRI, lower respiratory infection

^1^ Univariable logistic regression models were constructed for each predictor variable to obtain the odds of being an IFS case (as compared to IFP)

^2^ Logistic regression models were constructed with each pre-specified risk factor variable and adjustment for age to obtain the odds of being an IFS case as compared to IFP.

^3^ Logistic regression models were constructed with each pre-specified risk factor variable and multivariable adjustment for age at diagnosis, sex, hematologic malignancy (AML, ALL, MDS, MM, other) and transplant status (no transplant, transplant <1 year before fungal diagnosis, transplant >1 year before fungal diagnosis) to obtain the odds of being an IFS case as compared to IFP.

# eTable 2

Characteristics of IFS and IFP cases in sensitivity analysis (all proven cases diagnosed between 2014 and 2024)

| Characteristics | Invasive Fungal Sinusitis (n=23) | Invasive Fungal Pneumonia (n=22) |
| --- | --- | --- |
| Demographics |  |  |
| Age at IFD diagnosis  (years), median (IQR) ^4^ | 61 (54 – 68) | 59 (49 – 66) |
| Time from HM diagnosis to IFD (days), median (IQR) | 193 (42 – 1123) | 317.5 (94 – 937.3) |
| Male, n (%) | 12 (52.2) | 15 (68.2) |
| Hematologic malignancy, n (%) |  |  |
| Acute myeloid leukemia | 17 (73.9) | 17 (77.3) |
| Chronic lymphocytic  leukemia | 0 (0.0) | 2 (9.1) |
| Multiple myeloma | 1 (4.3) | 0 (0.0) |
| Other | 3 (13.0) | 3 (13.6) |
| Myelofibrosis | 1 (4.3) | 0 (0.0) |
| Non-Hodgkin lymphoma | 1 (4.3) | 0 (0.0) |
| Hematopoietic cell transplantation, n (%) | 12 (52.2) | 7 (31.8) |
| Comorbidities, n (%) |  |  |
| Diabetes mellitus | 6 (26.1) | 4 (18.2) |
| Underlying lung disease | 9 (39.1) | 2 (9.1) |
| Asthma | 3 (13.0) | 0 (0.0) |
| Bronchiectasis | 5 (21.7) | 2 (9.1) |
| COPD | 1 (4.3) | 0 (0.0) |
| Hypogammaglobulinemia | 11 (47.8) | 7 (31.8) |
| Viral URI or LRI | 4 (17.4) | 0 (0.0) |
| Chronic rhinosinusitis | 4 (17.4) | 1 (4.5) |
| Sinonasal anatomical  defect | 8 (34.8) | 1 (4.5) |
| Prior sinus surgery | 3 (13.0) | 0 (0.0) |
| Medications, n (%) |  |  |
| IVIG | 1 (4.3) | 2 (9.1) |
| Nasal steroids or  decongestants | 4 (17.4) | 5 (22.7) |
| Steroid inhaler | 4 (17.4) | 0 (0.0) |
| Oncologic treatment, n (%) |  |  |
| Systemic steroids | 6 (26.1) | 7 (31.8) |
| Antimetabolites | 20 (87.0) | 18 (81.8) |
| T-cell suppressants | 1 (4.3) | 1 (4.5) |
| GVHD in the last 60 days, n (%) | 4 (17.4) | 5 (22.7) |
| Spread of infection, n (%) |  |  |
| Localized | 11 (47.8) | 16 (72.7) |
| Disseminated | 12 (52.2) | 6 (27.3) |
| Azole prophylaxis at time of diagnosis, n (%) | 2 (8.7) | 6 (27.3) |
| Labs at time of IFD diagnosis, n (%) |  |  |
| ANC <500 | 18 (78.3) | 14 (63.6) |
| Any ANC <500 cells/μ in prior 6 months | 16 (69.6) | 20 (90.9) |
| Serum (1,3)-ß-D-glucan positive, n (%) | 5 (21.7) | 8 (36.4) |
| Galactomannan index positive, n (%) | 1 (4.3) | 5 (22.7) |
| Mucorales PCR positive, n (%) | 1 (4.3) | 4 (18.2) |
| Fungus identified, n (%) |  |  |
| *Aspergillus* species | 4 (17.4) | 6 (27.3) |
| Mucorales | 5 (21.7) | 8 (36.4) |
| *Curvularia* | 2 (8.7) | 0 (0.0) |
| *Fusarium* species | 5 (21.7) | 2 (9.1) |
| Hyaline mold, species not  identified | 1 (4.3) | 4 (18.2) |
| *Scedosporium* spp. | 1 (4.3) | 0 (0.0) |
| No observable species | 4 (17.4) | 2 (9.1) |
| Other | 1 (4.3) | 0 (0.0) |
| Time from IFD diagnosis to last follow-up (days), median (IQR) | 295 (31 – 670) | 189 (49.3 – 1124.8) |
| Time from IFD diagnosis to death (days), median (IQR) | 76 (25 – 303) | 109 (29 - 273) |
| Overall mortality at end of follow-up, n (%) | 15 (65.2) | 17 (77.3) |

Abbreviations: CI, confidence interval; IQR, interquartile range; COPD, chronic obstructive pulmonary disease; URI, upper respiratory infection; LRI, lower respiratory infection; IVIG, intravenous immunoglobulin; HSCT, hematopoietic stem cell transplant; GVHD, graft-versus-host disease; ANC, absolute neutrophil count; PCR, polymerase chain reaction; IFD: invasive fungal disease

# eTable 3

Age-adjusted and multivariable-adjusted logistic regression odds ratios for select risk factors differentiating IFS and IFP cases included in sensitivity analysis (all proven cases diagnosed between 2014 and 2024)

| Characteristic | Age-Adjusted Odds Ratio (95% CI) ^1^ | P-value | Multivariable-Adjusted Odds Ratio (95% CI) ^2^ | P-value |
| --- | --- | --- | --- | --- |
| Underlying lung disease | **6.23 (1.16 – 33.59)** | **0.03** | 5.74 (0.98 – 33.67) | 0.053 |
| Viral URI or LRI | *Did not converge* | -- | *Did not converge* | -- |
| Chronic rhinosinusitis | 4.59 (0.47 – 45.05) | 0.20 | 3.54 (0.28 – 43.87) | 0.33 |
| Sinonasal anatomical defect ^4^ | **13.92 (1.46 – 132.54)** | **0.02** | **14.38 (1.34 – 154.26)** | **0.03** |
| Prior sinus surgery | *Did not converge* | -- | *Did not converge* | -- |

Abbreviations: CI, confidence interval; URI, upper respiratory infection; LRI, lower respiratory infection

^1^ Logistic regression models were constructed with each pre-specified risk factor variable and adjustment for age to obtain the odds of being an IFS as compared to IFP.

^2^ Logistic regression models were constructed with each pre-specified risk factor variable and multivariable adjustment for age at diagnosis, sex, hematologic malignancy (AML, ALL, MDS, MM, other) and transplant status (no transplant, transplant <1 year before fungal diagnosis, transplant >1 year before fungal diagnosis) to obtain the odds of being an IFS case as compared to IFP.
